# Association between Take-Out Food Consumption and Obesity among Chinese University Students: A Cross-Sectional Study

**DOI:** 10.3390/ijerph16061071

**Published:** 2019-03-25

**Authors:** Yuhe Jiang, Junbo Wang, Shaowei Wu, Nan Li, Yiming Wang, Jiarui Liu, Xinran Xu, Zonghan He, Yawen Cheng, Xueqing Zeng, Bingwei Wang, Chenyu Zhang, Miao Zhao, Zhijie Su, Bingbing Guo, Wenzhong Yang, Ruimao Zheng

**Affiliations:** 1 Department of Stomatology, Health Science Center, Peking University, Beijing 100191, China; 1510303106@pku.edu.cn (Y.J.); wang-ym@pku.edu.cn (Y.W.); 1510303126@pku.edu.cn (X.X.); 1510303103@pku.edu.cn (Z.H.); chengyawen521@126.com (Y.C.); 1510303124@pku.edu.cn (X.Z.); 2 Department of Anatomy, Histology and Embryology, Health Science Center, Peking University, Beijing 100191, China; jbwang@bjmu.edu.cn (J.W.); ljr9210909@163.com (J.L.); bwwang@bjmu.edu.cn (B.W.); zcyfighting@163.com (C.Z.); MiaoZhao@bjmu.edu.cn (M.Z.); suzhijie@bjmu.edu.cn (Z.S.); guobingb@bjmu.edu.cn (B.G.); yangwenzhong@pku.edu.cn (W.Y.); 3 Department of Occupational and Enviromental Health Science, Health Science Center, Peking University, Beijing 100191, China; shaowei_wu@bjmu.edu.cn; 4 Institute of Reproductive and Child Health/Ministry of Health Key Laboratory of Reproductive Health and Department of Epidemiology and Biostatistics, School of Public Health, Peking University Health Science Center, Beijing 100191, China; linan01@pku.edu.cn; 5 Key Laboratory for Neuroscience of National Health Commission, Beijing 100191, China; 6 Neuroscience Research Institute, Peking University, Beijing 100191, China; 7 Key Laboratory for Neuroscience of Ministry of Education, Beijing 100191, China

**Keywords:** obesity, take-out food consumption, taste preference, major category, degree level

## Abstract

Background: The frequency of take-out food consumption has increased rapidly among Chinese college students, which has contributed to high obesity prevalence. However, the relationships between take-out food consumption, body mass index (BMI), and other individual factors influencing eating behavior among college students are still unclear. This study explored the association of take-out food consumption with gender, BMI, physical activity, preference for high-fat and high-sugar (HFHS) food, major category, and degree level among Chinese college students. Methods: Cross-sectional data were collected from 1220 college students in Beijing, China, regarding information about take-out food consumption, physical activity, and preference for HFHS food using a self-reported questionnaire. The logistic linear regression model was used to analyze the association between take-out food consumption and personal and lifestyle characteristics. Results: Out of 1220 college students, 11.6% of college students were overweight or obese. Among the personal and lifestyle characteristics, high frequency of take-out food consumption was significantly associated with a non-medical major, high preference for HFHS food, degree level, and higher BMI, but not physical activity. Conclusion: Among Chinese college students, consumption of take-out food may be affected by major category, preference for HFHS food, degree level, and BMI. This could provide guidance on restrictions of high take-out food consumption, which contributes to high obesity prevalence and high risk for metabolic diseases.

## 1. Introduction

Obesity, as an emergent public health issue, is a predisposing factor for many chronic diseases [1,2,3,4,5], such as type 2 diabetes, cardiovascular diseases, respiratory diseases [6], musculoskeletal disorders, and various types of cancer. Currently, nearly one-third of adolescents are overweight or obese in China; the obesity prevalence rate has risen from 0.10% in 1976 to 8.50% in 2016 [7], which is a relative increase by a factor of 85.0.

The obesogenic environment contributes to obesity [8]. Several lines of study have shown that fast-food consumption is closely linked to higher obesity prevalence in some Western countries [9,10,11] and China [12,13]. Fast food, such as hamburgers, French fries, pizza, and soda, are generally regarded as energy-dense and low-nutrient food, and tend to be rich in sugar and saturated fat. The number of restaurants of a major fast-food provider, Kentucky Fried Chicken (KFC), has increased to over 4200 in more than 800 cities and towns in China [14]. Another fast-food provider, McDonald’s, has also opened over 2000 restaurants across China from 1984 to 2014 [15]. The annual sales for China’s fast food industry increased from 24.2 billion in 2002 [15] to 107.0 billion United States (U.S.) dollars in 2015 [16], which is an increase of over 340%.

Notably, there are approximately 587 million students in colleges and universities across the world in 2016 [17], and one-fifth of these students are Chinese. Young Chinese are facing a remarkable transformation in their diet structure, as China is under rapid economic transition. Of note, Chinese college students, as an important driving force in economic development, also face multiple stressors, such as academic pressure, employment pressure, and debt burden [18]. Furthermore, owing to the advantages of low prices, delivery efficiency, and convenience, take-out fast food is becoming popular among Chinese students. All of these factors and the obesogenic environment around universities appeared to promote energy intake and constrain energy expenditure of the students. In China, take-out food has been regarded as a major form of fast food; thus, studying the relationship between obesity frequency and take-out food consumption in Chinese universities is critical for identifying areas for the exploration. However, previous evidence regarding the associations between fast-food consumption and obesity in Chinese students, and research in this area in countries under rapid socioeconomic transitions such as China is still limited.

Fast-food consumption among students might be influenced by several individual factors, such as preference for high-fat and high-sugar (HFHS) food, degree level, medical education background, body mass index (BMI), physical activity, and gender. It has been reported that obese individuals showed significantly higher preference for take-out food [19]; thus, the palatable taste of take-out fast food possibly contributes to obesity incidence [20]. However, it is unknown whether preference for HFHS food may affect the frequency of take-out food consumption among Chinese college students. A study showed that graduate students have more sedentary time, less spare time, and less physical activity, since graduate students have heavier academic pressure than undergraduate students in China [21]. In addition to degree level, a report suggested that medical students may be considered as a subpopulation with better health consciousness than non-medical students [22], which may lead to lower consumption of take-out food among medical students. However, until recently, no study has investigated whether these factors are associated with the frequency of take-out consumption between graduate and undergraduate students, or medical and non-medical students. Besides, some reports showed that female college students may have higher health consciousness, healthier dietary habits, and may pay more attention to their BMI in comparison to male students [23,24,25], and male college students are more susceptible to overweight and obesity than females [26]. Nevertheless, there is little research available regarding the associations of take-out food consumption with gender among Chinese college students.

The purpose of this study was to provide a more refined understanding on take-out food consumption in Chinese college students, and assess the influences of personal and lifestyle characteristics, including preference for HFHS food, medical education background (major category), degree level, BMI status, and gender on take-out food consumption.

## 2. Materials and Methods

### 2.1. Ethics Statement

The study was conducted between September and December in 2016 in China. Participants provided verbal informed consent, and participation was voluntary. No identifying information was collected. We tightly followed the guidance of ethics in this study to conduct analysis for research purposes, according to the guidelines laid down in the Declaration of Helsinki. All of the procedures involving human subjects were guided by the Ethics Committee of Peking University Health Science Center (PKUHSC) (LA2016113). This study met the criteria for exemption decided by the Institutional Review Board of PKUHSC. Public Health categorized this analysis as Not Human Subjects Research. Other investigators who are interested in accessing the dataset or replicating the analyses can contact the corresponding author.

### 2.2. Study Groups and Design

#### 2.2.1. Participants

This study is a cross-sectional study of college students enrolled in Peking University, China. All of the participants were selected through multistage stratified random cluster sampling, and participation was voluntary. This survey was conducted in 78 classes on two campuses, and 20 students were randomly investigated in each class. Investigators distributed questionnaires to each participant, and then collected them after completion. A total of 1560 students aged 18–26 years were selected, and 78.5% of them agreed to participate, while 335 students did not finish survey questions. Participants completed the questionnaire on gender, weight, height, major, degree level, grade, habit of eating take-out food, physical activity, frequency of take-out food, and preference for HFHS food, which was all self-reported. Five individuals were excluded due to abnormal BMI (BMI > 35). Therefore, the analytic sample was restricted to 1220 participants, including 528 males and 692 females.

#### 2.2.2. Assessment of Individuals’ BMI

The BMI (in kg/m^2^) of all of the participants was calculated as weight (kg) divided by the square of height (m^2^). According to criteria developed by the Chinese Working Group on Obesity in the International Life Science Association (WGOC) [27], we divided participants into four groups: underweight (BMI < 18.5), normal weight (18.5 ≤ BMI < 24), overweight (24 ≤ BMI < 28) and obese (BMI ≥ 28). We followed the WGOC standard, because Chinese people are usually at a higher risk of obesity-related diseases than Western people under the same BMI condition [28].

#### 2.2.3. Assessment of Take-Out Food Consumption and Preference for HFHS Food

According to previous studies [29,30], we defined take-out food as food items from fast-food outlets with convenient delivery service, carryout food options, and payment prior to the receipt of food. We collected information about the intake frequency of each meal (breakfast, lunch, dinner, or snack) during a seven-day period before the administration of the questionnaire, food types (staple food, vegetables, or meat) and duration of eating take-out food. Take-out food included seven categories of foods: (1) grains and cereals; (2) fruits; (3) vegetables; (4) milk and milk products; (5) meat and meat products; (6) fats, oils, and sugars; (7) and beverages [31]. Regarding the assessment of habit of eating take-out food, which meant a time period of eating take-out food at least once per week, we set four choices: (1) 0–3 months; (2) 4–6 months; (3) 7–12 months; and (4) 13–24 months.

In addition, we assessed all of the participants’ preference for HFHS food. A score scale of 1–4, representing no preference, little preference, moderate preference, and great preference, was used to evaluate the preference for four categories of HFHS foods: (1) meat pie, hamburger, or hot dog; (2) hot chips or French fries; (3) potato chips or savory snacks such as “Twisties”; and (4) biscuits, doughnuts, cake, or chocolate [32].

#### 2.2.4. Assessment of Physical Activity and Personal Information of the Study Participants

According to the Simple Physical Activity Questionnaire (SIMPAQ), we investigated the information about physical activity, which included time spent in bed, sedentary time, time spent standing, time spent walking, other physical activities, and exercise during one week [33]. Notably, light physical activity was excluded in our study, such as normal walking and use of bicycle. General information was also collected on the survey, such as age, gender (male, female), height, weight, major category (medical or non-medical), and grade (undergraduate level from first year to fifth year; graduate level from first year to third year). Medical students were those majoring in Basic Medical Sciences, Clinical Medical Sciences, Nursing, Pharmaceutical Sciences, Public Health, or Dentistry [34], and all of the other students were classified as non-medical students. The degree level was classified into two categories: undergraduate level (medical students, undergraduate years 1–5; non-medical students, undergraduate years 1–4), and graduate level (graduate years 1–3). At the end of survey, the investigator would record the corresponding class number and assign a random ordered number to the respondents from the same class.

#### 2.2.5. Adjustment Variables

Analyses were adjusted for grade (undergraduate years 1–5 and graduate years 1–3). Adjustment was also made for the habit of eating take-out food to help control for potential confounding from past eating habits. Since each participant was investigated independently, the influence between classmates should be zero when completing the survey.

### 2.3. Statistical Analysis

Participants included in the data analysis have complete information on take-out food intake and personal and lifestyle characteristics, and all of the individuals were divided into three subgroups by the frequency of take-out food consumption. Then, we examined the frequency of take-out food among college student subgroups divided by gender, major category, degree level, physical activity, preference for HFHS food, and BMI status. The Chi-square test was used to test the difference among the subgroups according to categorical variables, including gender, major category, degree level, physical activity, food preference, and BMI status.

In addition, in order to examine the association between take-out food consumption and personal and lifestyle characteristics, we used logistic linear regression models to calculate the odds ratios (OR) and 95% confidence intervals (CI). Preference for HFHS food, major category, degree level, and BMI status were included as independent variables in the regression models adjusted by gender and recent habit of eating take-out food (Model 1). On the basis of model 1, model 2 was further adjusted by physical activity to examine the association between take-out food consumption and personal and lifestyle characteristics. Since grade is highly correlated with degree level, we also conducted an analysis adjusted by gender, habit of eating take-out food, and grade (undergraduate years 1–5 and graduate years 1–3) (model 3). All of the statistical analyses were performed using IBM SPSS Statistics 24.0 (International Business Machines Corporation, Armonk, NY, USA), and statistical significance level was set at *p* < 0.05 (two-sided).

## 3. Results

### 3.1. Basic Anthropometric Characteristics

Descriptive characteristics of the participants are shown in Table 1. On the whole, there were 1220 students in the study population, with ages from 18 to 26. Among them, 56.7% were female students, 91.3% were undergraduate students, and 46.7% were medical students. In addition, 11.5% of these college students were overweight or obese, and the obesity prevalence was 3.5%. For the frequency of healthy behaviors, 48.4% students had high-level physical activity (>12 h per week) while 51.6% students had low-level exercise (≤12 h per week).

### 3.2. Assessment of the Frequency of Take-Out Food Consumption

With regard to take-out food consumption, obese participants had higher take-out food consumption, and medical students ate take-out food less frequently than non-medical students. Additionally, graduate students were more likely to eat take-out food than undergraduate students, and individuals with a higher preference for HFHS food were more likely to eat take-out food. Notably, there was no statistically significant association between physical activity and take-out food consumption among college students (Table 2).

### 3.3. Association between Take-Out Food Consumption and Personal and Lifestyle Characteristics

For factors influencing take-out food consumption, we found that degree level was also significantly associated with take-out food consumption (95% CI = 1.12–2.27, *p*-value = 0.01); preference for HFHS food had a positive effect on frequency of take-out food consumption (95% CI = 1.12–6.09, *p*-value < 0.001); high BMI was associated with an OR of 1.99 (95 % CI = 1.26–4.68, *p*-value = 0.04); and medical students had lower take-out food consumption than non-medical students (95% CI = 0.53–0.82, *p*-value < 0.001) (model 1, Table 3).

After further adjusting for physical activity, we obtained similar results as with model 1. Take-out food consumption was associated with a high degree level, high preference for HFHS food, high BMI, and medical background (model 2, Table 3). For model 3 (when control was made for gender, habit of eating take-out food, and grade), degree level became unassociated with frequency of take-out food consumption (95% CI = 0.60–1.78, *p* value = 0.90) (Table 3).

## 4. Discussion

In the present study, we provided a comprehensive evaluation of the factors influencing the frequency of take-out food consumption based on data from 1220 participants at Peking University, in Beijing China. We found that obesity prevalence among college students was lower than the national average [7]; high frequency of take-out food consumption was significantly associated with non-medical major, high preference for HFHS food, graduate study, and higher BMI in both male and female college students in our study participants. It is noteworthy that physical activity did not significantly affect take-out food consumption among college students.

Fast-food consumption has been studied in multiple areas. Researchers have investigated the association between fast-food consumption and educational level [35], socioeconomic status [36], dietary patterns [37], hypertension [35], and obesity prevalence [38,39,40,41] among adults in the community and among students in elementary school or college. For instance, by using data collected from Euromonitor’s Passport Global Market Information Database (GMID), 2012 edition, Roberto’s study investigated the effect of fast-food consumption on mean population BMI, and found evidence for the association between fast-food consumption and high BMI [40]. Mark’s research examining the association between reported fast-food habits and changes in body weight and insulin resistance among 3031 young adults in the United States showed that fast-food consumption has strong positive associations with weight gain and insulin resistance, which suggests that fast food increases the risk of obesity and type 2 diabetes [41]. In addition, Tambalis’s study used data from 177,091 Greek children and adolescents, and found that frequent fast-food consumption was associated with skipping breakfast and consuming sweets/candy, based on the EYZHN (National Action for Children’s Health) program [42]. On the basis of these results, many researchers have recommended further interventions to help children adopt healthier dietary habits.

A recent research study also examined the association between fast-food consumption and education level among 478 working adults in the United States [43]. The study used cross-sectional survey data collected from full-time and part-time employees of a large university located in the southeastern United States, and found that low educational attainment was related to a greater fast-food intake among women and men. For example, fast-food consumption was greatest in participants with no college education, and lowest in those with a graduate or professional degree. Specifically, males reported a greater fast-food consumption compared with females [43]. More recently, an elegantly designed cross-sectional study also found strong evidence for the influence of new media exposure to fast-food consumption among Chinese children and adolescents aged between six and 18 [44]. The study showed that watching online videos and playing computer games are behaviors associated with higher probabilities of eating at fast food restaurants in both rural and urban young residents, suggesting that new media exposure has an enormous effect on fast-food consumption in China [44]. In conclusion, previous work showed evidence for the influence of personal and lifestyle characteristics on fast-food consumption.

In our study, we also found some factors influencing take-out food consumption. Our contribution to the literature is that the frequency of take-out food consumption was significantly associated with major category, preference for HFHS food, degree level, and BMI among Chinese college students. College life is an important period for personal health. Compared to students from primary school or high school settings, college students have better economic conditions and can decide where to eat by themselves. They not only have meals in the school cafeteria, but also consume take-out food through an electronic trading platform, which provides a convenient delivery service. Since students in Peking University only have short winter and summer holidays (one month and two months, respectively), they are exposed to an obesogenic environment most of the time, such as MacDonald’s, KFC, and other fast-food restaurants around the university. In our study, students’ major category seems to be associated with their take-out food consumption. One potential explanation is that comparing to non-medical students, medical students are equipped with more professional medical knowledge and better health awareness, which are critical for avoiding consuming take-out food [22].

With regard to physical activity, vigorous-intensity exercise significantly promotes the development of good health consciousness in students, which is a potential factor for reducing take-out food consumption [45]. Surprisingly, based on our study, physical activity did not show a significant influence on take-out food consumption; this may have been attributed to the higher frequency of eating in the school cafeteria. Being characterized with cheap food and short waiting time, students prefer to have meals in school cafeteria rather than eating take-out food after vigorous-intensity exercise and low-intensity exercise. As the association between take-out food consumption and physical activity is still elusive, further research is needed to determine whether take-out food consumption is related to physical activity behaviors.

Another interesting finding in our study is that degree level was associated with take-out food consumption among Chinese college students. The odds of graduate students’ consumption of take-out food increased 1.19 times compared to undergraduate students. Graduate students tend to have higher academic pressure and less spare time; thus, they are more likely to consume take-out food due to the time saved. In this study, our analysis demonstrates that a high frequency of take-out food consumption was associated with a higher degree level among college students. Notably, there has been research showing that compared to those with high education levels, adults with low education levels have worse health consciousness [46], which may promote them to eat more low-quality food with high amounts of fat, cholesterol, saturated fatty acids, sugar, alcohol, and sodium. It suggests that working pressure and health consciousness may play important roles in influencing take-out food consumption. Therefore, our finding further suggests that an education-based strategy on the enhancement of health consciousness may serve as an intervention mean to restrict take-out food consumption. Importantly, reducing the number of take-out food restaurants around university may also contribute to the restriction on fast-food consumption.

There are several strengths in our study. To the best of our knowledge, this study is the first university-level study to investigate the associations between take-out food consumption and personal and lifestyle characteristics, including gender, preference for HFHS food, degree level, major category, and BMI status. Compared to Westerners, the risk of obesity-related diseases is usually higher among Chinese under the same BMI condition. Therefore, we categorized BMI according to the WGOC recommendation criteria. We also utilized a randomization design to support the validity of our results. Additionally, adjustment for individual histories of eating take-out food helps control the potential confounding from past eating habits.

Our study also has several limitations. The major limitations are the representativeness of our sample and the generalizability of our findings. Our data are not representative of the national college student population, and thus its generalizability may be limited. Given the geographical heterogeneity in economic development and nutritional environments, examining regional variations in take-out food consumption and its associations with health-related factors (e.g., BMI, physical activity, and preference of HFHS food) and demographic factors (e.g., gender, major category, and educational level) are encouraged in future research. Second, all of the data were self-reported by students, and therefore may be subject to potential recall bias. Third, our study lacks a measure of individual economic condition, which was presumably highly correlated with the frequency of take-out food consumption. Finally, the cross-sectional nature of our data does not support causal inferences between take-out food consumption and personal and lifestyle characteristics, which limits our study’s ability to guide practical interventions.

## 5. Conclusions

Our findings support that a high frequency of take-out food consumption was significantly associated with high preference for HFHS food, degree level, non-medical major, and high BMI. Based on our findings, we suggest that health-promoting activities should be directed to the students with unhealthy lifestyles, and should be particularly focused on decreasing the consumption of take-out food as well as improving medical knowledge [35]. Considering that take-out food consumption could be linked to adverse health outcomes through plausible mechanisms, further research is necessary to investigate the unhealthy consequences of rapidly increasing take-out food consumption, especially in the countries experiencing rapid economic growth. Most importantly, although the causes of obesity are multifaceted, public health measures to limit take-out food consumption in college students may be warranted. Such measures could include nutrition education campaigns, legislation to regulate the marketing of take-out food, and the elimination of take-out food from schools.

## Figures and Tables

**Table 1:** Basic information for the study population. BMI: body mass index.

Variable	Overall (N = 1220)		Male (N = 528)		Female (N = 692)	
	No.	(%)	No.	(%)	No.	(%)
Age						
18–23	1149	94.2	485	91.9	664	96.0
≥24	71	5.8	43	9.1	28	4.0
Body Mass Index						
Underweight	214	17.5	44	8.3	170	24.6
Normal weight	865	70.9	379	71.8	486	70.2
Overweight	98	8.0	77	14.6	21	3.0
Obese	43	3.5	28	5.3	15	2.2
Major						
Medical	570	46.7	241	45.6	329	47.5
Non-medical	650	53.3	287	54.4	363	52.5
Educational level						
Undergraduate	1114	91.3	471	89.2	643	92.9
Graduate	106	9.7	57	10.8	49	7.1

**Table 2:** Number of individuals across personal and lifestyle characteristics as well as categories of take-out food consumption. HFHS: high fat and high sugar.

Characteristics	Take-Out Food Consumption								*p*
	Overall		Tertile 1 (4–5 times/week)		Tertile 2 (6–8 times/week)		Tertile 3 (9–15 times/week)		
	No.	(%)	No.	(%)	No.	(%)	No.	(%)	
**Total**	1220	100	443	36.3	428	35.1	349	28.6	
**Gender**									
Men	529	43.4	174	14.3	191	15.7	164	13.4	0.08 *
Women	691	56.6	269	22.0	237	19.4	185	15.2	
**Major**									
Non-medical students	650	53.3	211	17.3	222	18.2	217	17.8	<0.001 ***
Medical students	570	46.7	232	19.0	206	16.9	132	10.8	
**Degree level**									
Undergraduate	1114	91.3	422	34.6	383	31.4	309	25.3	0.001 **
Graduate	106	8.7	21	1.7	45	3.7	40	3.3	
**Physical activity**									
Low (≦12 h/week)	629	51.6	233	19.1	215	17.6	181	14.8	0.78
High (>12h/week)	591	48.4	210	17.2	213	17.5	168	13.8	
**Preference for HFHS food**									
Low (2–6)	441	36.1	188	15.4	155	12.7	98	8.0	<0.001 ***
Moderate (7–9)	388	31.9	142	11.6	143	11.7	103	8.4	
High (10–16)	391	32.0	113	9.3	130	10.7	148	12.1	
**BMI**									
Underweight	214	17.5	71	5.8	71	5.8	72	2.9	0.02
Normal weight	865	70.9	296	24.3	291	23.9	278	22.7	
Overweight	98	8.1	31	2.5	32	2.6	35	2.9	
Obese	43	3.5	7	0.6	10	0.8	26	2.1	

*: *p* < 0.05, **: *p* < 0.01, ***: *p* < 0.001.

**Table 3:** Odds ratios (ORs) of take-out food consumption associated with personal and lifestyle characteristics.

Characteristic	Model 1 ^1^		Model 2 ^2^		Model 3 ^3^	
	OR (95% CI)	*p-*Value ^3^	OR (95% CI)	*p-*Value	OR (95% CI)	*p-*Value
**Preference for HFHS food**						
Low (2–6)	1.00	—	1.00	—	1.00	—
Moderate (7–9)	1.39 (1.14–3.05)	0.001 **	1.56 (1.20–2.04)	0.001 **	1.58 (1.22–2.04)	<0.001 ***
High (10–16)	2.61 (1.12–6.09)	<0.001 ***	2.08 (1.58–2.70)	<0.001 ***	2.27 (1.61–2.78)	<0.001 ***
**Major**						
Non-medical students	1.00	—	1.00	—	1.00	—
Medical students	0.66 (0.53–0.82)	<0.001 ***	0.66 (0.57–0.81)	<0.001 ***	0.67 (0.53–0.81)	<0.001 ***
**Degree level**						
Undergraduate	1.00	—	1.00	—	1.00	—
Graduate	1.61 (1.12–2.27)	0.01 *	2.19 (1.39–3.43)	0.009 **	1.03 (0.60–1.78)	0.90
**BMI**						
Underweight	1.00	—	1.00	—	1.00	—
Normal weight	1.41 (0.84–3.21)	0.15	1.19 (0.54–2.62)	0.67	1.67 (0.84–3.33)	0.17
Overweight	1.62 (0.89–2.91)	0.09	1.69 (0.84–3.03)	0.16	1.69 (0.94–3.13)	0.06
Obese	1.99 (1.26–4.68)	0.04 *	2.02 (1.78–5.46)	0.02 *	2.26 (1.98–5.83)	0.01 *

*: *p* < 0.05, **: *p* < 0.01, ***: *p* < 0.001. **^1^** Model 1 was adjusted by gender and habit of eating take-out food; **^2^** Model 2 was adjusted by gender, habit of eating take-out food, and physical activity; ^**3**^ Model 3 was adjusted by gender, habit of eating take-out food, and grade.
